# The Applications of Health Informatics in Medical Tourism Industry of Iran

**Published:** 2017-08

**Authors:** Peyman REZAEI-HACHESU, Reza SAFDARI, Marjan GHAZISAEEDI, Taha SAMAD-SOLTANI

**Affiliations:** 1. Dept. of Health Information Technology, School of Management and Medical Informatics, Tabriz University of Medical Sciences, Tabriz, Iran; 2. Iranian Center of Excellence in Health Management, School of Management and Medical Informatics, Tabriz University of Medical Sciences, Tabriz, Iran; 3. Dept. of Health Information Technology, School of Allied Medical Sciences, Tehran University of Medical Sciences, Tehran, Iran

## Dear Editor-in-Chief

Some countries in Africa and the Middle East, such as Iran, have potential advantages in medical tourism industry, including low-cost and high-quality services, competent physicians and marginal natural and historical attractions. A systematic and comprehensive program ensure the development of medical tourism industry (1) and lack of systems to collect information about the health status of tourists, inefficient information systems, and legal problems was discussed as major challenges in policymaking process. Telecommunication and Informatics have important role in establishment a strong infrastructure to improve political, social and legal issues in this industry (2). Health informatics is an interdisciplinary field of information science, computer and health care that deals with resources, equipment and methods to improve acquisition, storage, retrieval and use of health and biomedicine information (3). In the following, important potential aspects of health informatics application in medical tourism will be discussed:

- *Electronic Health Card* has been adopted by all European Union countries being operational in supporting the medical tourism and increasing patients’ degree of decision. The information on the electronic health card is in two categories. The administrative category contains necessary data about insurance status, rights to be treated abroad, and prescriptions. The medical category is optional and contains only information to which the patient allowed, such as drug information, demographic and clinical information. Tourists can also attach their own health documentation, such as a disease diary (4).
- *Electronic Health Record (EHR)* is information technology systems based on a technological and informational infrastructure from the medical organizations. The development of EHR platform and application or Medical Registries at patient’s level provides the positive aspects of IT and ICT introduction in healthcare especially in medical tourism (4). EHR system will lead to an increase in the capabilities of medical tourism facilities to attract medical tourists from developed countries. Tourist’s personal medical information can be accessed globally over a network by using EHR.
- *Telemedicine* refers to use of information technology to treat or monitor patients remotely by ICT from simplest tools to modern technologies. Telemedicine is becoming common in areas where physicians are scarce (5). The use of telemedicine in medical tourism as well as the healthcare manager’s intentions toward the cost of the service models and valuing health promotion and prevention. In the medical tourism industry, a patient who lives in another country can obtain valid information about his health status by tele-consultation with a physician and select the best treatment options for their travel plan.
- *Virtual social network* is one of the oldest and most capable ICT applications related to tourism. The purpose of all these systems is to help tourists, before, during and after travel to get better experience (6). Virtual medical tourism communities will provide a strong foundation with which to bring up communication among and between travelers and the healthcare provider. Web forums, social groups and channels, question and answer systems and group instant messaging are the potential tools for virtualization of communication.
- *Data mining and knowledge discovery* was described as the process of analyzing large data sets to survey and extract earlier unidentified patterns, styles, and relationships to produce knowledge for decision making. Combined with analytics tools, an EMR system provides valuable knowledge that doctors can use to analysis patient problems and patients can learn about economical treatment for his/her diseases and conditions without leaving home.
- *Medical Tourism Recommender Systems(RS) and Decision support systems(DSS)*, have been widely applied as tools for reducing the information overload and providing travel recommendations to tourists. RSs are currently being applied in many various domains by using many different functionalities and platforms such as web-based, mobile, intelligent, DSS, ranking systems, scheduling systems, routing apps, positioning systems and etc. most of these systems have portions are associated with artificial intelligence.

Recommendation and suggestion rules are the knowledge of the systems.

Finally, it suggests that at the same time with development of medical tourism industries in Iran, the potential power of medical informatics applications to be applied to boost integration, interoperability, and knowledge extraction tasks in low, middle and high level of decision making.
